# The origin of great ape gestural forms

**DOI:** 10.1111/brv.13136

**Published:** 2024-08-27

**Authors:** Kirsty E. Graham, Federico Rossano, Richard T. Moore

**Affiliations:** ^1^ School of Psychology & Neuroscience University of St Andrews St Mary's St Mary's Quad, South St St Andrews KY16 9JP UK; ^2^ Department of Psychology Hunter College 695 Park Avenue New York NY 10065 USA; ^3^ Department of Cognitive Science University of California San Diego 9500 Gilman Drive La Jolla 92093 California USA; ^4^ Department of Philosophy University of Warwick Coventry CV4 7AL UK

**Keywords:** great ape gestures, meaning, communication, language evolution, pragmatics, adaptation, exaptation

## Abstract

Two views claim to account for the origins of great ape gestural forms. On the Leipzig view, gestural forms are ontogenetically ritualised from action sequences between pairs of individuals. On the St Andrews view, gestures are the product of natural selection for shared gestural forms. The Leipzig view predicts within‐ and between‐group differences between gestural forms that arise as a product of learning in ontogeny. The St Andrews view predicts universal gestural forms comprehensible within and between species that arise because gestural forms were a target of natural selection. We reject both accounts and propose an alternative “recruitment view” of the origins of great ape gestures. According to the recruitment view, great ape gestures recruit features of their existing behavioural repertoire for communicative purposes. Their gestures inherit their communicative functions from visual (and sometimes tactile) presentations of familiar and easily recognisable action schemas and states and parts of the body. To the extent that great ape species possess similar bodies, this predicts mutual comprehensibility within and between species – but without supposing that gestural forms were themselves targets of natural selection. Additionally, we locate great ape gestural communication within a pragmatic framework that is continuous with human communication, and make testable predications for adjudicating between the three alternative views. We propose that the recruitment view best explains existing data, and does so within a mechanistic framework that emphasises continuity between human and non‐human great ape communication.

## I. INTRODUCTION

In the study of the origins of great ape gestural repertoires, two views predominate. On the first (historically older) view, which we call the “Leipzig view”, great ape gestures are a product of *ontogenetic ritualisation* (Tomasello *et al*., 1985, 1997*b*; Tomasello, Gust & Frost, 1989). On the ritualisation view, non‐human great apes learn to use gestures; but not in the same way that children learn to use words. Rather, gestural signs emerge through repeated dyadic interactions between pairs of individuals as non‐communicative actions become progressively conventionalised, turning into signals that acquire communicative properties. Gestural signs are therefore acquired in ontogeny through processes of interaction, and are often specific to pairs of communicators.

According to the rival “St Andrews view”, the great ape gestural repertoire is not learned in ontogeny but is a product of our biological inheritance (Byrne *et al*., 2017). On this view, gestures are taken to be species‐general communicative signals that emerged under natural selection for particular gestural forms. Because of our shared genetic inheritance, the five great ape species (including humans) are taken to have substantially overlapping gestural repertoires (Kersken *et al*., 2019; Graham & Hobaiter, 2023). The last common ancestor (LCA) of humans and orang‐utans (*Pongo pygmaeus*; the great ape species to which we are most distantly related) had a small gestural repertoire that incrementally expanded during evolutionary history. The result is much larger but still overlapping gestural repertoires in chimpanzees (*Pan troglodytes*) and bonobos (*Pan paniscus*); and a potentially larger but still genetically inherited repertoire in humans – although this may have fallen into disuse following the emergence of natural languages.

Herein we propose a new, hybrid view, which we call the “recruitment view”. According to this view, great apes recruit visually salient and largely shared postures [intentional movements that correspond to (parts of) familiar actions schemas, for example reaching up towards another in preparation for climbing up onto them], bodily parts (locations on an individual's body, e.g. shoulders to be groomed), and states of the body (temporary changes in physiology, e.g. erect genitalia or piloerect fur) for communicative purposes, showing these to express their communicative goals (Grice, 1957). The recruitment view works slightly differently in the three different types of gesture we describe. In the first kind (Section IV.1), the feature recruited for communicative means is (part of) a familiar action schema. In the second type (Section IV.2) it is a body part or a state of the body. In the third case (Section IV.3), a tactile gesture corresponds to a mechanically effective version of the same action (e.g. a gentle nudge is used communicatively to move someone, instead of pushing them into place more forcefully). Nonetheless, in each case, the feature recruited for communicative use is a familiar and contextually interpretable feature of non‐communicative great ape interactions.

We retain from both the Leipzig and St Andrews views the idea that great ape gestures are often related to familiar action sequences, and that the meanings of gestures can typically be specified in terms of the actions they are used to solicit (e.g. Hobaiter & Byrne, 2014). However, we argue against the claim of the Leipzig view that the communicative function of gestures arises through repeated dyadic interactions that shape the gestural form, and we reject the St Andrews view that the cross‐species similarity of gestures is a product of natural selection for gestural forms. Rather, we argue that gestural forms are recruited from existing non‐communicative behaviours, action sequences, and bodily states, and then used for communicative purposes. To the extent that the bodies of great apes are largely similar, and that they perform similar actions in pursuit of similar goals, great ape gestures may be understood by members of their own and other great ape species without the need for any history of interaction and ritualisation between pairs of individuals. Thus the recruitment view predicts mutual comprehension between strangers even without a shared learning history. To the extent that great ape gestures inherit their communicative functions from species‐general action schemas and familiar bodily states, they may also be comprehensible by humans (even those who lack experience of observing great ape interactions) (Graham & Hobaiter, 2023).

By explaining the mutual comprehensibility of great ape gestures in this way, we avoid what we take to be an unwarranted adaptationist tendency of the St Andrews view, while still accounting for the similarity in form and function of many great ape gestures. We support our proposal with illustrative examples of the visual features of gestures from the great ape repertoire, and make explicit, testable predictions about all three views. Finally, we elaborate a more detailed account of the cognitive mechanisms that support gestural communication on the view that we defend. We do not claim that our account extends to an explanation of great ape vocal communication, but it may be a suitable model for characterising the gestural interactions of other species.

Before developing our own account we consider the existing explanations in more detail.

## II. THE LEIPZIG VIEW: ONTOGENETIC RITUALISATION

The first influential account of the origins of great ape gestures was developed by Tomasello *et al*. (1985, 1989, 1997*b*) and Call & Tomasello (2007) using insights drawn from Tinbergen (1951) and Plooij (1978). On the ontogenetic ritualisation view, there are two varieties of ape gesture: “intention movement signals” and “attention getters” (Tomasello *et al*., 1989). Intention movement signals are ritualised from non‐communicative behaviours and emerge spontaneously in dyadic interactions between individuals who interact frequently. Processes of ritualisation can arise as follows. Where individual A performs action α, and individual B responds by performing β, after repeated interactions partners can anticipate one another's behaviour. Thus, B might start to perform β before A has finished performing α. Since B can now grasp A's intended action when she performs only part of α, over time A need no longer perform all of α when wanting to solicit B to perform β. Instead, A can perform only the first part of α and rely on B anticipating what she wants and producing β in response. Thus, A can produce the first part of α as a sign, with the communicative function of soliciting response β from B. In this account, gestures emerge through repeated interactions, wherein both signaller and recipient learn that an intended outcome can be achieved by this particular truncated action, now a gesture. The intended outcome is the starting point and gestures are learned through this ritualisation process as a means to achieving the intended outcome more efficiently.

There is empirical support for the hypothesis that at least some great ape gestures can be ontogenetically ritualised. For example, a study of mother–infant interactions in captive bonobos showed that some gestures learned by infant bonobos are ritualised from non‐communicative actions related to the initiation of particular movements (Halina, Rossano & Tomasello, 2013). A similar pattern can be observed in wild infant chimpanzees in the development of the gestures they use to elicit joint locomotion with their mothers (Fröhlich, Wittig & Pika, 2016) – although since publishing the latter study, some of its authors have re‐interpreted their findings as evidence that social experience is required for the emergence of gesture, rather than ontogenetic ritualisation (Pika & Fröhlich, 2019).

The ontogenetic ritualisation hypothesis makes certain predictions that differentiate it from alternative views of the origins of the great ape gestures (Table 1). For example, since gestural signs are ritualised from repeated interactions between pairs of individuals, there may be significant variation between the gestures used by different pairs of individuals: so‐called “idiosyncratic gestures”. The simplest way in which this might occur is that individuals ritualise their gestures to different points in the action schema, with some gestures thus representing the action more or less completely. Alternatively, suppose that two individuals in a group engage in a variant of a common action sequence that differs from the equivalent action performed by other members of that group. While the other members of the group perform action α to elicit β, one individual in this group performs α_1_. Here the ritualisation hypothesis predicts that the idiosyncratic individuals may produce a different gesture to elicit β from other members of the same group. Individuals may also use different gestures for the same function when communicating with individuals with whom they have a different history of interactions. Early evidence for idiosyncratic gestures came from small captive populations of chimpanzees where only certain individuals used certain gestures in certain study periods (Tomasello *et al*., 1985, 1989, 1997*b*). Many studies do report rare gesture types used by one or few individuals, which may be considered idiosyncratic although these represent one or two gesture types in repertoires of dozens of gesture types (e.g. Call & Tomasello, 2007; Graham, Furuichi & Byrne, 2017; Hobaiter & Byrne, 2011*b*).

**Table 1:** Predictions of the two previous hypotheses claiming to account for the origins of great ape gestural forms, and of the Recruitment hypothesis presented herein.

	Leipzig view	St Andrews view	Recruitment hypothesis
Repertoire overlap or variability among individuals, communities, or species	Predicts moderate but sometimes limited overlap across communities and variability of individual repertoires. The amount of variability within a community will depend on the frequency of interactions among individuals. Predicts significantly less intragroup than intergroup variability. Across communities some gestures may not be mutually comprehensible if they have been heavily ritualised and the underlying behaviours are no longer recognisable.	Predicts high overlap of repertoires across individuals, communities, and species (with some repertoire pruning, see *Gesture emergence/acquisition*), as the entire gesture repertoire is biologically inherited. Since gestural forms are part of a shared genetic inheritance, for gestures in the repertoire of both species this view predicts mutual comprehension between species.	Predicts high overlap of repertoires across individuals, communities, and species, because they have similar bodies and similar goals. Additionally, where gestures are not the same, there should be substantial mutual comprehensibility of unfamiliar gestures. There will be mutual comprehension between species insofar as embodied action schemas match.
Gesture emergence/acquisition	Individuals learn through repeated interactions with the same social partners. All gestures are learned through social interaction.	Individuals are equipped with the capacity to use all gesture types in the repertoire. As juveniles they go through “repertoire pruning” to settle on a somewhat smaller final repertoire.	Minimal learning is needed to explain communication between unfamiliar individuals because gesture forms are try‐out variants of familiar behavioural action schemas. Additional gestures may be acquired, but shared learning history is unnecessary for mutual comprehension of most gestures.
Flexibility of gestures across contexts and/or meanings	Ritualised gestures should be tied to a specific meaning but can be used in different contexts. So, a begging gesture will mean “give me” but it can be used in multiple contexts.	No explicit predictions.	Predicts flexibility of gestures across contexts and meanings. Mutual comprehension is made possible by a common ground of shared bodies against which gestures are improvised. Thus, we predict more pragmatic flexibility.
Redundancy; multiple gestures for the same meaning	Because of ritualisation, individuals may have several gestures for the same function. This is likely true across dyads, but it has also been shown to be true within dyads (see Halina *et al*., 2013). Redundancy comes about because a partner might stop responding to some signals and so the communicator might try to elicit a response by producing a different behaviour.	No explicit predictions although it should predict a limited amount of redundancy because of phylogenetic ritualisation.	Predicts redundancy as a feature of the many actions that individuals have available to them to express their intended goals. This is because multiple behaviours may be associated with the performance of the same types of goal‐directed activity.
Resemblance of gestures to actions/outcomes	Gestures may resemble actions (or parts of actions) related to their intended outcome. However, they may have been ritualised such that they are no longer recognisably related to body schemas from which they are ritualised.	Gestures may resemble parts of actions involved in achieving the outcome because they have been phylogenetically ritualised from such actions. However, they may have been ritualised such that they are no longer recognisable as such.	Gestures should resemble actions (or parts of actions) related to their intended outcome, as well as ostensively presented bodily states, because the interpretability of gestures is derived from these visually salient forms.

Tomasello and others also describe a class of gestures that they call “attention getters”, in which great apes intentionally make a noise or perform an action that solicits attention to themselves from their interlocutor (Tomasello, 2008). This can be done for a variety of reasons, including as a way of soliciting attention to features of the gesturing individuals that are not under their voluntary control – for example, piloerect fur, threat displays, and erect genitalia. Thus, the performance of attention getters serves to amplify the significance of these states and presents them to interlocutors with a communicative function. Nonetheless, evidence for the existence of attention getters is mixed. Hobaiter & Byrne (2014) argue that no gestures in the repertoire that they analysed were performed exclusively to solicit attention – they achieved other behavioural goals too. For that reason, we do not here follow Tomasello in assuming that great ape gestures can be described in terms of the dichotomy of attention‐getters and intention movement signals.

## III. THE ST ANDREWS VIEW: A BIOLOGICALLY INHERITED COMMUNICATIVE REPERTOIRE

As part of an ambitious programme to record and analyse the gestural repertoires of all four non‐human great ape species, and in response to the ritualisation hypothesis defended by the Leipzig School, Byrne and colleagues formulated the St Andrews explanation of gestural origins. A key premise of their account is that claims about the idiosyncrasy of pair‐specific gestures between great apes were driven largely by the small sizes of early studies. They argue that with more sustained, larger scale observations gestural repertoires are found to overlap (almost) entirely across the members of each great ape species.

Moreover, they argue, there is large overlap in gestural repertoires across great ape species. Gestures used by *Pan* (bonobos and chimpanzees) (Graham *et al*., 2018) are also used by both *Gorilla gorilla* (gorillas) and *Pongo* (orang‐utans), and even in humans (Hobaiter & Byrne, 2011*b*; Kersken *et al*., 2019; Graham & Hobaiter, 2023). Given that the gestures used by non‐human great apes constitute only a small subset (70–90) of the 1000+ gestures estimated to be morphologically possible, Byrne *et al*. (2017, p. 761) state that this convergence is “unlikely to be a coincidence”. Rather, it is taken to be an indicator of a common biological descent. Thus, on this hypothesis, particular gestural forms are argued to have undergone selection processes, perhaps *via* phylogenetic ritualisation, through which they have become associated with particular communicative functions. This account therefore reflects the view that in the evolutionary sciences, the primary mode of explanation should be the identification of processes of natural selection through which behavioural traits have emerged (R.W. Byrne, personal communication). It also treats great ape gestures in the same manner that their vocalisations have traditionally been studied – namely, as products of natural selection.

On the St Andrews view, individuals also produce their gestures with certain goals, but the gestures with which they seek these goals are part of a genetically inherited repertoire. Ontogeny is not taken to be irrelevant to the process of gesture acquisition. Since many gestures have multiple uses, juvenile learners may still need to experiment with gestures to identify the best ways in which to use them. Hobaiter & Byrne (2011*a*) found that young chimpanzees produce sequences of gestures in pursuit of a single goal. As they age, individuals make more frequent use of the most effective gestures, potentially due to the pruning of less‐effective ones, with adult individuals using fewer gesture sequences than immature individuals. Nonetheless, the St Andrews view holds that both the gestural forms and semantic features (i.e. communicative functions) of great ape gestures are an unlearned product of our biological inheritance.

Byrne and colleagues are right that in the evolutionary sciences, the default hypothesis is to look for adaptive explanations of behaviour. Often this explanatory strategy takes the form of researchers identifying species‐general behavioural traits and hypothesising explanations with ecological reasons that made these traits adaptive in a species' evolutionary history, and then arguing that these traits became targets of natural selection processes. The same strategy can also be used to explain the appearance of similar traits in closely related species. Traits that appear in neighbouring clades are hypothesised to be the result of processes of adaptation by natural selection in an ancestral group. However, *contra* Byrne and colleagues, we need not assume that gestural forms were themselves the targets of natural selection, even though great ape bodies are products of many complex and overlapping adaptive processes. We think it more appropriate to characterise them as exaptations (Lloyd, 2021). On the view that we propose, the great ape gestural repertoire consists of acts of showing – that is, of communicative presentations of familiar postures, and parts and states of the body. In such cases, the goals of these communicative acts (i.e. the messages with which they are produced) are closely associated with features of the shown postures, parts, and states, making the utterances easily interpretable even in interactions between unfamiliar individuals. We need not think of these bodily action schemas, parts, and states as having undergone selection for their communicative function. Rather, because they are already present in the great ape non‐communicative repertoire they can be recruited for communicative purposes. For example, erect genitalia can be shown (or “addressed”; Moore, 2017*b*) to interlocutors to communicate sexual interest, or familiar parts of action sequences shown to others (by virtue of being addressed to them) to solicit responses appropriate to those actions.

## IV. EMPIRICAL EVIDENCE FOR THE RECRUITMENT VIEW

The above sections set out our theoretical rationale for rejecting both the Leipzig and St Andrews accounts of the origins of great ape gestures. We now illustrate how the positive account we propose is consistent with existing empirical studies of great ape gesture forms. We present evidence that some great ape gestures resemble the actions about which they are used to communicate (Section IV.1), and that others show bodily parts and states of the body that are connected to the gestures' communicative functions (Section IV.2). In both types of these gestures, their function and interpretability are driven by showing salient visual features that correspond to the communicated behaviour. In a third kind of gesture we describe (Section IV.3), interpretation is supported by not visual but tactile features. In Section V, we try to disambiguate further the account that we have proposed from existing views, by making some predictions about the different commitments that might be entailed by the Leipzig, St Andrews, and Recruitment views of great ape gestural origins. We do this to sketch ways in which further empirical evidence might be collected to establish which view gives the best account of gestural origins.

In the examples below, we elaborate on the kinds of resemblance that we think are fundamental to great ape gestural communication and illustrate our claim with examples.

### (1) Presenting action schemas

Many great ape gestures visually recall the actions with which their communicative function is associated, often by visually resembling parts of an action schema. Typically, these gestures correspond to what Tomasello (2008) calls “intention movement signals” – although for reasons described in Section II, we do not adopt his distinction between attention getters and intention movement signals. We recognise that there are limits to what should count as resemblance, since otherwise the term may be emptied of explanatory power – everything resembles everything else, in some respect. The notion of resemblance used herein is not fully worked out, but it is comparable to the idea of resemblance adopted in other accounts of iconic features of great ape communication (e.g. Scott‐Phillips & Heintz, 2023) where authors appealed to the idea of a resemblance between the form and meaning of a gesture. The idea that we wish to convey is that, for a large set of chimpanzee gestures, the form of the gesture corresponds to part of an action sequence that could serve as a meaning to the fulfillment of the gesturer's goal, and that this similarity is salient to the intended recipient of the gesture.

**Fig. 1:**
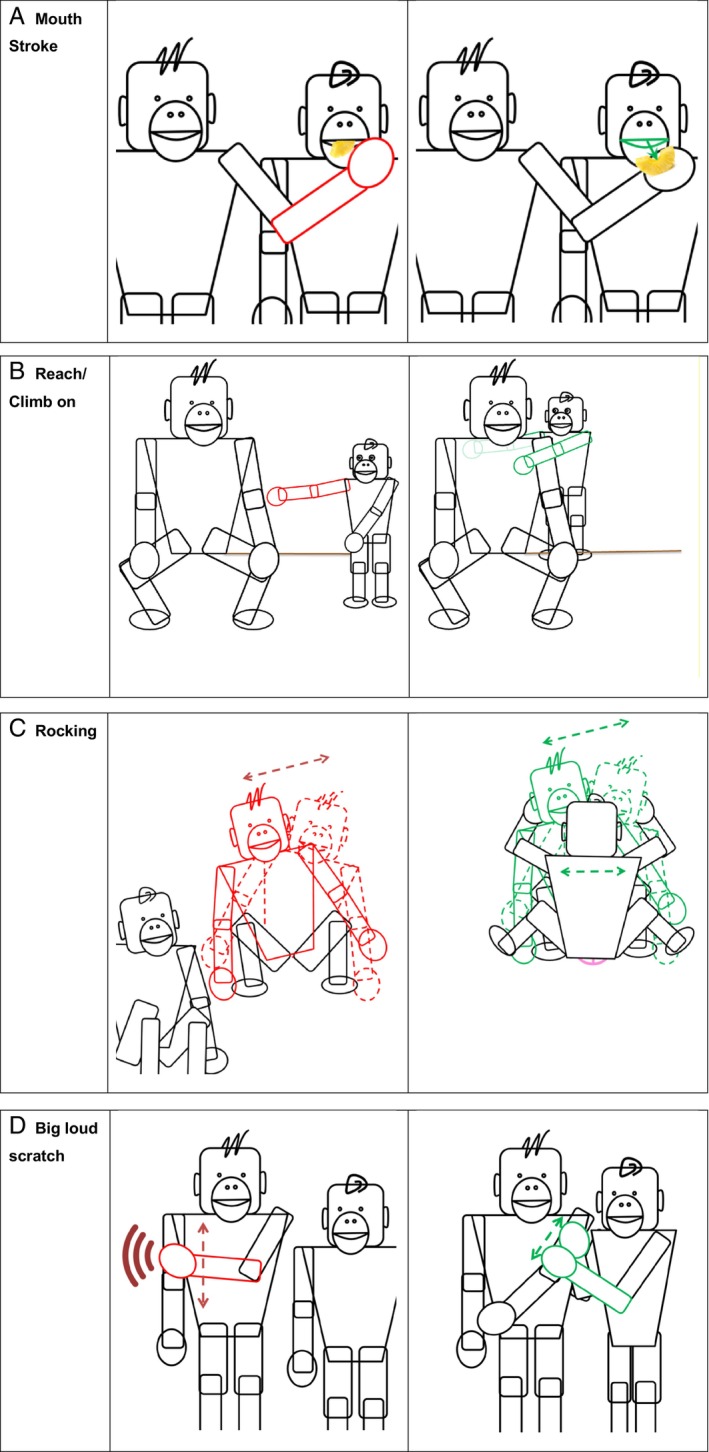
A selection of chimpanzee and bonobo gestures and the outcomes that they achieve, illustrating visual resemblance between the gesture action and its outcome. In this and the following legends, percentages indicate the proportion of times a gesture is used with a particular function. Many (although not all) great ape gestures are used with multiple communicative functions. (A) In the “Mouth stroke” gesture, a “signaller's palm and fingers are repeatedly run over the mouth area of the recipient” (Hobaiter & Byrne, 2011*b*, Table 1, p. 755). Bonobos and chimpanzees use the “Mouth stroke” to request “Acquire object/food” (100% and 87%, respectively) (Graham *et al*., 2018). (B) The “Reach/Climb on” gesture is performed by extending an arm “to the recipient with hand in an open, palm upwards, [downwards, or sideways] position” (Hobaiter & Byrne, 2011*b*, Table 1, p. 755). In bonobos and chimpanzees, the “Reach” gesture is the most commonly used gesture to achieve the outcome “Climb on you” (Hobaiter & Byrne, 2014). (C) To perform the “Rocking” gesture, sitting bonobos “rock forward and back or side to side, repeated[ly]” (Graham *et al*., 2017, supplementary material 1, p. 1). This is done to request “Initiate copulation” (54%) or “Initiate genito‐genital (GG) rubbing” (46%) (Graham *et al*., 2018). (D) The “Big loud scratch” gesture is performed by a “loud exaggerated scratching movement on the signaller's own body” (Hobaiter & Byrne, 2011*b*, Table 1, p. 753). It is used by bonobos and chimpanzees as a request to “Initiate grooming” (100% and 82%, respectively) (Graham *et al*., 2018).

Our point is best illustrated with examples. For example, in making begging gestures, chimpanzees often hold their hands towards the mouth of the individual from whom they are begging, so that their interlocutor might drop or spit some food from their own mouths into the chimpanzee's hand (Fig. 1A). The familiar chimpanzee palm‐up begging gesture thus resembles the act of taking food from or reaching for the mouth of a peer, since in the process of taking food from that mouth the taker's hand occupies a similar position to the one used in the begging gesture. Similarly, a young bonobo's arms‐up “Climb on” gesture in which the gesturer's hands reach upwards towards its mother's chest or back, visually resembles the act of her climbing up onto her mother (see Fig. 1B); and in the “Rocking” gesture (Fig. 1C), bonobos reproduce movements that occur during sexual intercourse. These resemblances are likely salient to the intended recipients of gestures. In these kinds of gestures, we can think of great apes as showing their interlocutor's action schemas or body postures that are means to the performance of the actions that the gestures are used to convey. These gestures thus visually resemble the actions that they are used to communicate about. They can be used communicatively by being addressed to the attention of an interlocutor, typically by using eye contact and producing the gesture in the direction of the intended recipient (Gómez, 1994, 1996; Moore, 2016, 2017*b*), in a manner intended to elicit a response to the gestures. These gestures are typically used to solicit actions that will contribute to the fulfilment of the gesturer's goal. For example, in the case of a begging gesture, an individual might let food drop from their mouth into the outstretched hand of the gesturer. In the case of the “Climb on” gesture, the recipient might extend a part of their own body towards the gesturer to facilitate their efforts to climb onto them.

The Leipzig view posits that the communicative function of these gestures arises through a process of ontogenetic ritualisation – although as signs are ritualised, the familiar action sequences from which gestures are ritualised may become harder to recognise. The St Andrews view invokes phylogenetic ritualisation to explain any resemblance between gestural form and function. However, we argue that since these gestures visually resemble elements of the action sequences that they are used to initiate they need neither be learned nor explained as a product of phylogenetic ritualisation. Rather, by virtue of their possessing similar bodies, and recognising the same actions, individuals may recognise the functions of gestures as connected to familiar action sequences, even when gestures are performed by unfamiliar individuals, and without any shared learning history. As a result, these gestures may be easily interpretable as having semantic properties closely connected to the actions or parts of action sequences that they resemble. These gestures can also be performed by individuals who recognise an association between their own body postures and the performance of certain kinds of goal‐directed actions. For this to be possible, users need some basic familiarity with/experience of the kinds of bodies and action schemas depicted in the gestural performances. However, since relevant non‐communicative experience will make the functions of gestures easily recognisable, it is unnecessary to suppose that the communicative function of these gestures is secured by ritualisation in either ontogeny or phylogeny.

To propose that great ape gestures acquire their semantic properties by resembling the actions with which they are associated is not to claim that these gestures are iconic in the fullest sense of the word. Thus, while some have argued that some great apes do produce and understand iconic gestures (Tanner & Byrne, 1996; Russon & Andrews, 2010; Perlman, Tanner & King, 2012; Douglas & Moscovice, 2015; Genty & Zuberbühler, 2015; see also Scott‐Phillips & Heintz, 2023), we do not (and need not) claim this. Iconic gestures are often characterised as those which are produced with the intention visually to recreate an action schema for communicative purposes (Moore, 2014). While great ape gestures are both intentionally produced and visually resemble action schemas, we do not suppose that great apes need be reflectively aware of this resemblance for their gestures to be successful. That is, we do not suppose that gesture selection is guided by a metarepresentational process in which the gesturer intends that her audience recognise that her gesture resembles an action that is connected to her communicative goal. Rather, it may be that, because these body postures are already associated with the performance of certain actions (through the repeated experience of their co‐occurrence), recreating them becomes an intuitive, unreflective choice for agents engaged in the gesture selection process. Nonetheless, there is clearly an analogy between our own account of the origins of great ape gestures and Charles Sanders Peirce's account of icons – signs whose interpretation is mediated by a visual likeness (Peirce, 1897/1903; Scott‐Phillips & Heintz, 2023). On our account it is visual (and sometimes tactile) resemblance that forges the association between a gesture and its function, rather than a history of ritualisation.

### (2) Showing body parts

**Fig. 2:**
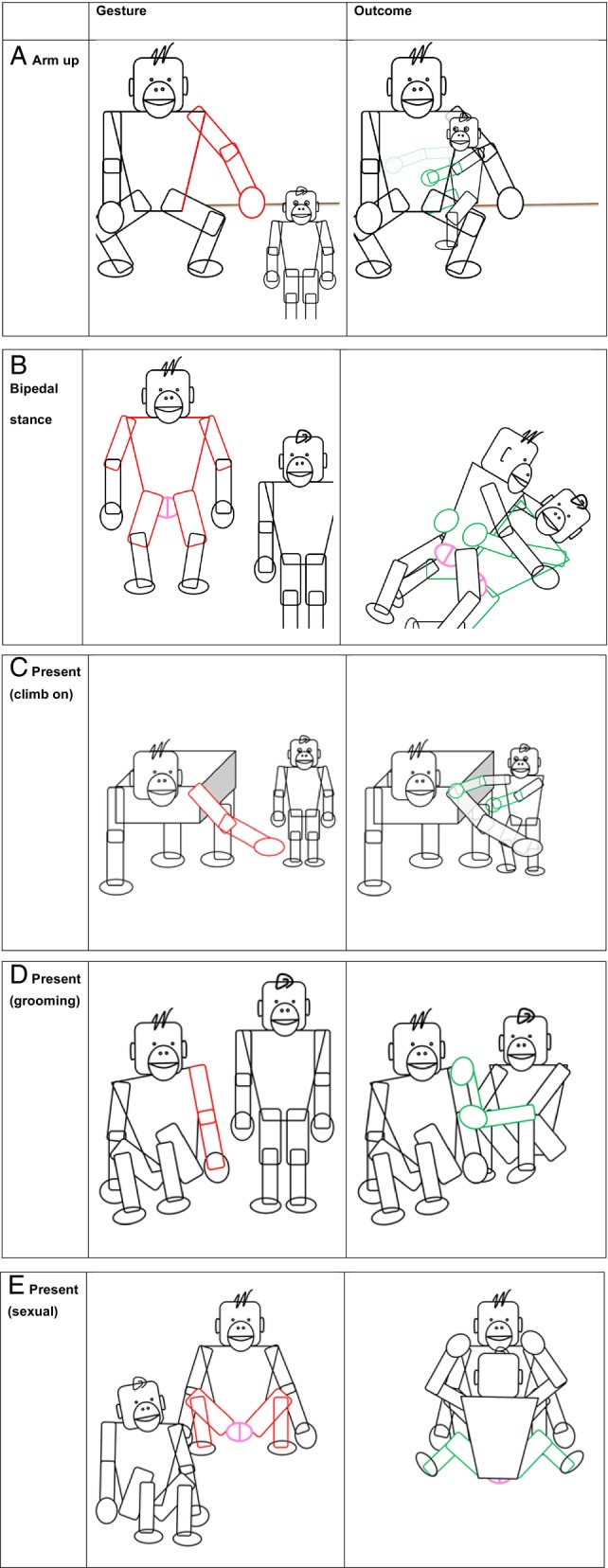
A selection of gestures that show the part of the body with which the recipient should interact. (A) The “Arm up” gesture deploys extended “straight arm(s) out to side and away from body” (Graham *et al*., 2017, supplementary material 1, p. 1). It is used by bonobos to request “Initiate contact” (80%) and “Climb on me” (20%) (Graham *et al*., 2018). (B) In the “Bipedal stance” gesture, apes stand bipedally, arms out to the side, and with their back arched, to present their exposed genitalia (Graham *et al*., 2017). It is used by bonobos to request “Initiate copulation” (50%) and “Initiate genito‐genital (GG) rubbing” (50%) (Graham *et al*., 2018). (C) In the “Present (climb on)” gesture, an “arm or leg is extended to young recipient in order to facilitate them climbing onto the signaller's body (normally mother to infant)” (Hobaiter & Byrne, 2011*b*, Table 1, p. 756). Both chimpanzees and bonobos use this gesture to request “Climb on me” (100% for both species) (Graham *et al*., 2018). (D) In the “Present (grooming)” gesture, the body is moved to “deliberately expose an area to the recipient's attention which is immediately followed by grooming of the area” (Hobaiter & Byrne, 2011*b*, Table 1, p. 756). Bonobos and chimpanzees both use this to request “Initiate grooming” (100% for both species) (Graham *et al*., 2018). (E) In the “Present (sexual)” gesture, the signaller “sits and spreads their limbs displaying their genital swelling or erect penis” (Graham *et al*., 2017, supplementary material 1, p. 4). This gesture is used by bonobos to request “Initiate genito‐genital (GG) rubbing” (64%) and “Initiate copulation” (36%), and by chimpanzees to request “Initiate copulation” (74%) (Graham *et al*., 2018).

In a second type of great ape gesture, the signaller shows a body part to the recipient – in the past these have been circularly defined by the outcome that they achieve: “Present (Climb on)”, “Present (grooming)”, “Present (sexual)” [sometimes split as “Present (genitals forward)” and “Present (genitals backward)”] (Fig. 2C–E). It may be more informative to look at the body part that is presented and the body part that the recipient interacts with. We have also identified the “Arm up” gesture as one that potentially presents part of the body (i.e. by exposing the individual's front and side) for the recipient to interact with, as the main outcome was “Initiate contact” (Fig. 2A). “Bipedal stance” could also be considered as a modified version of “Present (sexual)” with the signaller standing while presenting their full front and genitals to the recipient (Fig. 2B).

This set of gestures may function like Tomasello's “attention getters” – although we do not claim that the sole function of such gestures is to solicit attention in a non‐specific way. Rather, we propose that the contents of attention‐soliciting messages are provided by implicit reference to accompanying contextual features of the utterances – potentially including objects, locations, and the concurrent presence of bodily states that are not under intentional control, but to which great apes draw their interlocutors' attention by showing or addressing (Gómez, 1994, 1996; Moore, 2016, 2017*b*) them to others. Some of the states to which individuals solicit the attention of their interlocutor can be described by reference to what Bar‐On (2013) calls expressive behaviours. These are behaviours that, whether or not they are themselves under intentional control, provide insights into the state of mind of an individual, in ways that could provide evidence of her accompanying goals. For example, piloerect fur provides insights into the agitated state of an individual's mind. When great apes solicit attention to their bodily states or parts in the way described here, they do so to draw attention to some further goal, which is implicitly expressed through the bodily state to which they are drawing attention – for example, piloerect fur, erect genitalia, or body parts that they wish to be groomed. While actions that draw attention to locations and objects do not recruit states or parts of the gesturer's body, they may nonetheless function in the same way. Since the objects and locations are already likely to be salient as affording certain actions, attention‐soliciting behaviours that draw attention to them will inherit their interpretability from the significance of these accompanying features.

As with gestures that inherit their form from action schemas (Section IV.1), we do not need to suppose that this repertoire of communicative acts was a target of natural selection for communicative purposes (although in some cases it may have been). Rather, individuals recruit existing features of their bodies or their environment by drawing attention to them for communicative purposes. Sometimes these features will be products of natural selection – as in the case of piloerect fur, erect genitalia, or emotional facial expressions. But we need not posit any further natural selection process to explain how these states are recruited by great apes for intentional communication. For example, a shoulder can be shown to another for the purpose of grooming, or (as in Section IV.1) a hand can be held towards another's mouth to request food, but we need not suppose that either presentation of the shoulder or the extension of the hand have undergone natural selection for communicative purposes. The existing body parts and bodily states are already significant to potential interlocutors, and individuals need only draw attention to their presence to recruit these behavioural states for communicative ends. When these gestures are addressed to others in order to solicit from them certain actions, this combination of actions makes them communicative: they are being ostensively shown to an audience, with a communicative goal (Gómez, 1994, 1996; Moore, 2017, 2017). In addition, while these processes of recruitment could be learned, there is no reason to suppose they must have been learned for their communicative functions to be interpretable. The behaviours through which non‐human great apes express their communicative intentions are already likely to be universally understood, at least where these behaviours are part of the species‐typical repertoire. For example, with background knowledge/experience of non‐communicative social life in great ape communities, backs may be recognised as things that can be scratched, groomed, or climbed upon; and genitals as parts of the body to be engaged with sexually. As a result, communicative acts that recruit behaviours to express the communicative goals with which they are already correlated are readily interpretable.

While we have categorised separately gestures that involve the recreation of parts of action sequences (Section IV.1) those that involve showing parts or states of the body (Section IV.2), and those involving tactile resemblance (Section IV.3), these categories do not need to be mutually exclusive. Some gestures in the great ape repertoire may incorporate elements of both. For example, the “Big loud scratch” gesture (Fig. 1D) potentially involves the recreation of both elements of the act of scratching and draws attention to the area of the body its producer wants the interlocutor to scratch. In principle, features of different gesture types could be combined freely, and their combinations would serve further to facilitate comprehension.

### (3) Directional contact gestures

**Fig. 3:**
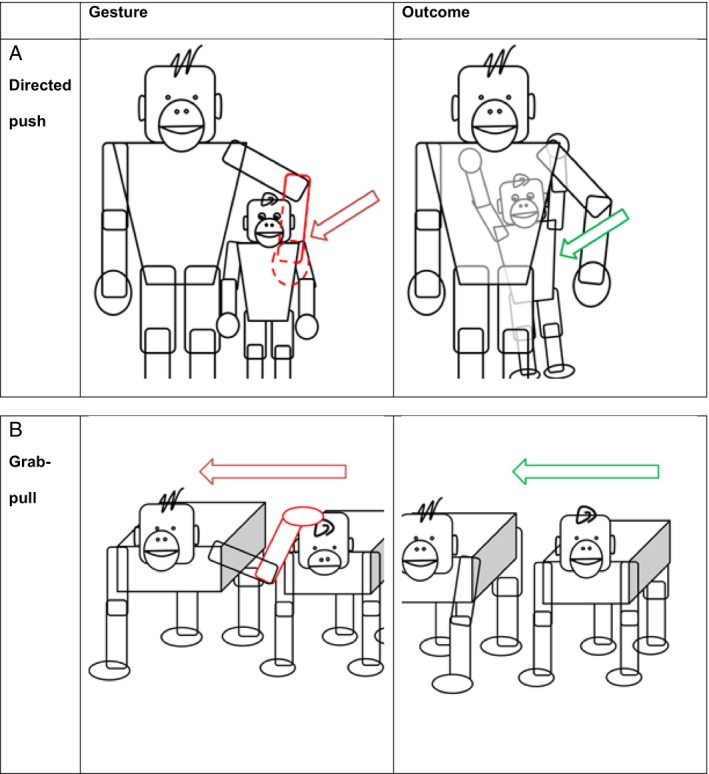
Two gestures that direct the recipient towards a certain location. (A) The “Directed push” gesture is a “light short non‐effective push that indicates a direction of desired movement, immediately followed by the recipient moving as indicated” (Hobaiter & Byrne, 2011*b*, Table 1, p. 753). It is used by bonobos to request “Climb on me” (100%), and by chimpanzees to request “Reposition” (50%), “Move closer” (21%), and “Climb on me” (15%) (Graham *et al*., 2018). (B) In the “Grab‐pull” gesture, a “closed hand contact is maintained and a force exerted to move the recipient from their current position” (Hobaiter & Byrne, 2011*b*, Table 1, p. 754). The gesture is used by bonobos to request “Follow me” (58%), “Reposition (21%)” and “Climb on me” (10%) and by chimpanzees to request a peer to “Move closer” (31%), “Climb on me” (25%) and “Contact” (13%) (Graham *et al*., 2018).

“Push” (or “Directed push”) (Fig. 3A) is a contact gesture where the signaller applies a force towards the recipient's body. Here the resemblance that facilitates understanding of the communicative function of a gesture may not only be visual but also tactile. A gesture can both visually resemble the act of manoeuvring another and also feel like it. For example, the “Pull” gesture (or “Grab‐pull”) (Fig. 3B) is a contact gesture where the signaller applies a force away from the recipient's body. Both gestures likely use applied force to indicate a direction of movement for the recipient. For bonobos, “Directed push” was used to mean “Climb on me” in all successful instances, and for chimpanzees the top three meanings were “Reposition”, “Move closer”, and “Climb on me” (Fig. 3A). By their definition, “Directed push” was used when the recipient moved in the direction of the force applied by the signaller. But to test this properly, future research would need to assess direction of force and direction of recipient movement. “Grab‐pull” was coded without considering directionality, but the meanings suggest movement related to the gesturing: for bonobos, the top three meanings were “Follow me”, “Reposition”, and “Climb on me”; for chimpanzees, the top three meanings were “Move closer”, “Climb on me”, and “Contact” (Fig. 3B).

## V. THE ROLE OF LEARNING

Current theories of gesture development and use in non‐human primates (mostly based on great ape data) suffer from challenges posed by empirical evidence. The Leipzig view requires extensive learning within repeated dyadic interactions, so that gestures can only be used inflexibly – both with respect to their function, and with respect to the likelihood that gestures will be understood by individuals who were not part of the dyad from which the gestures emerged (Table 1). This should in principle lead to an individual potentially producing different gesture types to achieve the same outcome while interacting with different individuals. It also predicts limited repertoire overlap across communities and across species because of the unpredictability of the ritualisation process. However, in practice there is large overlap in gestural repertoires across individuals, communities, and species. The number of signals produced to achieve the same outcome is also rather limited and not clearly partner dependent. Therefore, the amount of learning necessary for mutually comprehensible gestural communication between great apes seems to be less than the Leipzig view would predict. The St Andrews view on the other hand predicts limited redundancy in terms of the number of gestures associated with a specific meaning (Table 1). Such redundancy nonetheless exists. The St Andrews view also predicts only a limited form of learning, in the form of correctly mapping innate functions (i.e. meanings) to innate gestural forms, and where this mostly takes the form of giving up the use of gestures that do not solicit intended responses. Nonetheless, we know that there is some level of learning in great ape communication at least in the context of joint‐locomotion and carry signals (Halina *et al*., 2013; Fröhlich *et al*., 2016). New gestures can be and are learned.

The Recruitment view presents a model that can account for current empirical evidence of gestural communication in great apes without requiring *ad hoc* explanations for counter empirical evidence. It also requires less learning than the Leipzig view and yet accounts for both some degree of variability in the repertoire and a large amount of overlap across communities and even species. This view also avoids making the unwarranted assumption that gestural forms were themselves targets of natural selection, and it presents claims that are easily testable and potentially falsifiable.

The account of great ape gestural origins that we develop here has some similarities with another influential recent account of the origins of the great ape behavioural repertoire –Tennie's *latent solutions* approach (Tennie, Call & Tomasello, 2009; Tennie *et al*., 2020). On both views, individual great apes spontaneously invent their own solutions to the challenges they encounter. In the case of gestural communication, individuals create their own gestures using gestural forms that are salient to them because they incorporate either familiar actions schemas or body parts/states. By virtue of their having similar bodies, the same forms are independently salient to all, because all individuals will associate (even if unreflectively) broadly the same actions with the same goals. As a result, they produce and understand one another's gestures with relative ease, and the gestures produced by different individuals are visually similar. This may be the case even if individuals do not select gestures because they are reflectively aware of the visual resemblance of their chosen gesture to the action specified in their intended message, and expect their interlocutor to be similarly aware of the resemblance. The chosen gestures might thus be described as “latent solutions” to the communicative challenges for which they are used (Tennie *et al*., 2020). Individuals could potentially invent gestures and still be understood because, by virtue of their shared body schemas, signallers and recipients associate the same postures and body movements with the same outcomes and goals.

This is not to say that there is no learning involved in the development of great ape gestural communication. As previously noted, some basic familiarity with bodies and actions schemas is required, and this may be acquired during ontogeny. Additionally, as Hobaiter & Byrne (2011*a*) described, there may be a period in which juveniles experiment with their gestures, before they become confident in using and understanding them. The social negotiation hypothesis (Pika & Fröhlich, 2019) posits that “gestures emerge from an exchange of social behaviours between interactants” (p. 557). While not full ontogenetic ritualisation, it requires that individuals actively engage in joint behaviours with others for gestures to emerge. In this account, individuals should only produce gestures to initiate behaviours that they themselves have already engaged in with others. This differs somewhat from our account, whereby having similar bodies and similar behaviours (as individuals) is sufficient for formulating intentions. Experience likely shapes gesturing preferences, but we would predict that individuals can use gestures to initiate behaviours with others prior to having experienced those behaviours with others. Behaviours that emerge later in development, such as sexual contact, could be used to test this prediction. Nevertheless, we share Pika & Fröhlich's (2019) view that learning need not take the form of any period of ontogenetic ritualisation.

We do not discount the possibility that great apes can learn new gestures – although this seems rare in captive individuals (Tennie, Call & Tomasello, 2012), possibly because they are not motivated to extend their communicative repertoire. If new gestures are learned though, we predict that their communicative functions should be tied to associated action schemas or body parts/states. Tennie's failed attempt to teach untrained chimpanzees new gestures notably involved arbitrary gestural forms (Tennie *et al*., 2012), which are particularly difficult for chimpanzees to learn (Bohn, Call & Tomasello, 2016; Berio & Moore, 2023).

For all that we think visual resemblance is central to (and perhaps necessary for) the function and interpretation of gestures, visual resemblance alone is likely insufficient for comprehension of novel gesture–action pairings. This is reflected in studies of iconicity that find low comprehension of novel gestures produced by humans (Bohn *et al*., 2016, 2020). In the case of gestures that correspond to parts of action schemas, we think resemblance is made apt to be recruited for communicative means through its familiarity with a common behaviour, and because that behaviour is *communicatively relevant* to some ongoing interaction. There may be many parts of actions that could be recruited for communication, but which relate to behaviours that are not communicatively relevant to the flow of great apes' social lives. In such cases, even visually salient gestural forms might be overlooked. Furthermore, even where gestures relate to communicatively relevant interactions, unenculturated great apes' generally poor social attention (especially towards humans; Berio & Moore, 2023) might constitute a further obstacle to successful interpretation. We think great apes' poor comprehension of both human pointing and iconic gesture is likely partly attributable to their inattention to particular features of human behaviour, including potentially the orienting features and shapes of gesturing human hands (Tramacere & Moore, 2020; Berio & Moore, 2023). This might also undermine their ability to interpret novel gestures produced by conspecifics.

Nonetheless, new gestures could potentially be innovated within groups of great apes, and we predict that these would be readily interpretable without a history of ritualisation. One way to test our hypothesis would be to observe whether newly seeded behaviours in a group (e.g. van Leeuwen, Cronin & Haun, 2014) also give rise to communicative interactions involving gestures over time. If they do, one could subsequently seek to determine whether there is some visual resemblance between the form and function of newly created gestures. We predict that where additional gestures are learned, these will recruit either familiar action schemas and/or body parts and states, and consequently that these gestures would be used and understood spontaneously by individuals familiar with the new behaviour.

We concede that new gestures could also be learned through a process of ontogenetic ritualisation, and we acknowledge that some elements of the repertoire of captive chimpanzees may be best explained by appeal to processes of ontogenetic ritualisation. Nonetheless, such cases should be relatively rare. The recruitment view predicts that the mutual comprehensibility of most gestures in the repertoires of great ape species does not depend upon any extended period of interaction in which ontogenetic ritualisation could take place. Rather, even unfamiliar individuals' gestures will be mutually comprehensible without a shared learning history, on account of the visual resemblance between gestural forms and the meanings with which gestures are used. It may also be that over time gestures initially used spontaneously become habitual, such that apes need not reinvent them on each occasion of use. But this is consistent with their initially being usable without any process of ritualisation.

## VI. EMBODIED GESTURAL COMMUNICATION AND LANGUAGE EVOLUTION

We believe a further advantage of the recruitment view is its potential to contribute to debates about language evolution, and particularly to “pragmatics‐first” accounts of language development (Tomasello, 2008; Scott‐Phillips, 2014; Moore, 2017*b*; Heintz & Scott‐Phillips, 2022; Bar‐On, 2024). Such accounts posit that the first natural languages emerged against a background of non‐verbal communicative interactions, and specifically between users who could act with and attribute communicative intentions.

The St Andrews view provides relatively little insight into the evolution of language, beyond predicting some overlap in the gestural repertoire of all great ape species. However, regarding the mechanics of production and comprehension, it presents a semantics‐first account of how communication works: great apes communicate using signs that acquired both their form and function from processes of natural selection. On such an account, there is no need for agents to be able to act with and attribute communicative intent since production and comprehension are under the control of natural selection. Thus, pragmatic interpretation may be limited to using contextual features of production to interpret a fixed repertoire of signs or calls (Wheeler & Fischer, 2012). This kind of contextual inference, however, is of the wrong kind to explain the evolution of language (Bar‐On & Moore, 2017; Moore, 2018; Bar‐On, 2021), because most theories of language evolution assume that our ancestors could act with and attribute communicative intentions prior to language development (e.g. Tomasello, 2008; Scott‐Phillips, 2015; Moore, 2018), and because knowing how to interpret signs across contexts is insufficient for acting with or interpreting communicative intent.

Proponents of the Leipzig view have argued that no great ape species act with and attribute communicative intentions, entailing that the socio‐cognitive abilities of ancestral hominins must have undergone substantial development before they could use and understand language (Tomasello, 2008; Scott‐Phillips, 2014; Scott‐Phillips & Heintz, 2023). Tomasello (2008) explicitly contrasts a view of communication *via* ontogenetic ritualisation with a view of communication via communicative intentions, and posits this as a major point of difference in the communicative abilities of humans and other great apes, explaining why only humans acquired language (see also Scott‐Phillips & Heintz, 2023). This view is problematic both because it is motivated by intellectualised and misleading philosophical accounts of the nature of cognition required for communication rather than by empirical data (Moore, 2017, 2017), and because there is clear empirical evidence that there are circumstances in which great apes can understand human communicative intentions (Lyn, Russell & Hopkins, 2010) including some forms of language (Savage‐Rumbaugh & Murphy, 1993; Truswell, 2017). The challenge of explaining enculturated great apes' comprehension of human communication therefore remains for both St Andrews and Leipzig views.

By contrast, the view we have presented is consistent with another possibility. On this view, all species of great ape are capable of acting with and attributing communicative intentions, albeit in restricted ways. Specifically, we argue that there are constraints on the kinds of communicative intentions that unenculturated great apes can interpret and attribute, because the natural repertoires of these species are heavily dependent upon the bodily expression of the gesturer's message, and the ways in which this visually salient bodily expression supports the interpretation of communicative goals. Where these perceptually salient, familiar behaviours and body postures are not present to support utterance interpretation, as in the case of pointing (which is highly ambiguous, since interpretable in multiple ways), comprehension may be difficult for non‐human great ape species (Tomasello, Call & Gluckman, 1997*a*; Moore, 2013*a*). It may be only after substantial training or exposure to human behaviours (e.g. through enculturation in infancy) that they become able to interpret the communicative goals behind a wider range of behaviours (Leavens, Hopkins & Bard, 2005; Leavens, 2006). This is consistent with the possibility that great apes in zoos and in the wild are poor at arbitrary, symbolic forms of communication (Bohn *et al*., 2016); but that they can acquire symbolic abilities through extended periods of enculturation, perhaps because of the ways in which enculturation changes great ape attention (Berio & Moore, 2023).

On our proposal, we can think of great apes as acting with and attributing communicative goals – albeit in ways that are limited in comparison to humans. There are various ways in which human and great ape communicative interactions are likely to differ. Some of these are already well established in the literature. For example, great apes may act primarily with only relatively simple kinds of “directive” communicative goals – for example, to make requests or give orders (Bullinger *et al*., 2011), and with utterances produced to inform others only relatively rarely (Zimmerman *et al*., 2009; Crockford *et al*., 2012). The contents of their goals may also be simpler than our own – for example, because they do not involve complex psychological states (Moore, 2017*b*), and because they lack the syntactic complexity of human communication (e.g. Rivas, 2005; Truswell, 2017). Here, we propose a further limitation on their communication interactions: while great apes may be able to act with and attribute communicative goals (Moore, 2016, 2017, 2017), their capacity for pragmatic interpretation is limited in comparison to ours, and is dependent upon the presence of salient and easily interpreted parts of action schemas, and parts and states of the body.

To justify this claim, more needs to be said about the way in which we conceive of ostensive‐inferential (or “Gricean”) communication – that is, communication that involves acting with and attributing communicative intentions (or “goals” – here we use the terms interchangeably). Consistent with others (e.g. Sperber, 2000; Csibra, 2010; Scott‐Phillips, 2014), we take ostensive‐inferential communication to be a process in which communicators produce utterances with certain goals, and ostensively address their gestures to the attention of their intended audience, for example by engaging them in eye contact, and directing their gestures to the interlocutors' attention (Gómez, 1996; Moore, 2017*b*). On the basis of being so addressed, audience members attempt to infer the goals with which their interlocutors are producing utterances (Csibra, 2010). However, as noted before, we differ from others in supposing that Gricean communication need not be socio‐cognitively demanding (Moore, 2016, 2017, 2017; see also Gómez, 1994). Once we accept that this process need not be socio‐cognitively demanding, there is good reason to believe that great apes can do this (Gómez, 1994; Moore, 2016, 2017, 2017).

With respect to the process of inferring communicative goals, following Mercier & Sperber (2017), we take the process of inference to be one in which an agent makes a judgement about some state based upon incomplete evidence. In the case of communication, inferring a gesturer's communicative goal will consist of making a judgement about what message they intend to communicate, based upon the combination of behaviours through which their intended message is expressed and addressed (Moore, 2017*b*). What we hypothesise herein is a constraint on the pragmatic interpretation abilities of great apes. While they may be able to attribute communicative goals to one another, they may only be able to do this where there is a strong perceptual resemblance between the gestures and the messages that the gestures are used to communicate, or where presented bodily states are easily interpretable.

This is consistent with the finding that apes may be able to interpret the intentions of their interlocutors based entirely on contextual features of the interaction, with gestures acting more as a prompt to elicit a response (Bohn *et al*., 2022), since there may be multiple behavioural and contextual sources from which evidence of a speaker's intentions is inferred. Nonetheless, our view suggests further predictions about the kinds of gestures that are likely to be interpreted by (unenculturated) great apes.

The hypothesis that great apes express their communicative goals by showing others postures and bodily states requires acknowledging that *showing* can be a form of Gricean communication. This claim is historically controversial, because Grice himself rejected it [Grice, 1989; see Moore (2017*c*), for discussion]. Nonetheless, philosophers in the Gricean tradition (Neale, 1992; Wharton, 2003; Moore, 2017*c*) now typically accept that showing can involve the production and interpretation of communicative intentions. For example, I may show you my black eye as a way of communicating that I have had a bad day, or my broken foot as a way of saying that I will not be playing football tonight.

We argue that great ape gestures regularly incorporate elements of showing – either in the form of shown action schemas, or parts and states of the body – and that both their communicative function and interpretability is derived from this. This is a point of contrast with human communication: unlike most human symbols, great ape gestures are not arbitrary (Moore, 2013*b*). A key step in the evolution of natural languages was likely the acquisition of an ability to use and interpret arbitrary, non‐iconic gestural forms. Interestingly, domestic dogs also fare much better at interpreting certain embodied gestures than physically similar non‐bodily signs (Moore *et al*., 2015). While enculturated great apes can learn to use symbols to communicate (Savage‐Rumbaugh & Murphy, 1993), such symbols are not a part of their ordinary phenotypic repertoire.

The view defended here thus generates a new set of predictions about the course of language evolution, identifying a key transition as the emergence of abilities for interpreting communicative intentions between a wider set of signs (see also Moore *et al*., 2015). Our proposal also has potential application for explaining communication in other species. It may be that what makes human communication unique is not a uniquely human ability to attribute communicative intentions, but rather our ability to interpret communicative intentions based on more limited evidence of communicative goals from the signaller. Since our approach can potentially contribute to explanations of communication in non‐ape species, and even has some commonalities with influential theories of the embodied nature of linguistic metaphors in human communication (Lakoff & Johnson, 2008), it might be thought of as a contribution to a general theory of the embodied basis of communication.

## VII. CONCLUSIONS

(1) Standard accounts of the origins of great ape gestural forms are unsatisfactory. The Leipzig view overestimates both the role of learning in the development of gestural forms, and the amount of within‐ and between‐group variation in signal use. Meanwhile, the St Andrews view assumes – without justification – that gestural forms were themselves targets of natural selection.
(2) Our novel “Recruitment view” explains the possibility of mutual comprehension within and between great ape populations (and potentially species) without learning, and without assuming that gestural forms are themselves a product of targeted selection. On this view, great apes communicate by showing visually salient body parts, postures, and states.
(3) In addition to sketching a novel mechanistic account of great ape gesture production and comprehension, we locate this communicative behaviour within a framework of pragmatic interpretation that is continuous with human communication. On this view, both humans and great apes can act with and attribute communicative intentions. Nonetheless, non‐human great apes may be strongly dependent upon the presence of visually salient and easily interpreted behaviours to facilitate their interpretation of others' communicative goals, in ways that humans are not.
(4) This framework for explaining gestural communication in great apes could potentially be applied to other species.
